# Establishment of Tumor Treating Fields Combined With Mild Hyperthermia as Novel Supporting Therapy for Pancreatic Cancer

**DOI:** 10.3389/fonc.2021.738801

**Published:** 2021-11-03

**Authors:** Liping Bai, Tobias Pfeifer, Wolfgang Gross, Carolina De La Torre, Shuyang Zhao, Li Liu, Michael Schaefer, Ingrid Herr

**Affiliations:** ^1^ Molecular OncoSurgery, Section Surgical Research, Department of General, Visceral and Transplantation Surgery, University of Heidelberg, Heidelberg, Germany; ^2^ Medical Research Center, Medical Faculty Mannheim, University of Heidelberg, Heidelberg, Germany; ^3^ Department of Hematology, Oncology and Rheumatology, Internal Medicine V, University Hospital of Heidelberg, Heidelberg, Germany

**Keywords:** pancreatic ductal adenocarcinoma, hyperthermia, tumor treating fields, alternative therapies, bioinformatics and computational biology

## Abstract

Pancreatic ductal adenocarcinoma (PDAC) is a highly malignant tumor with poor prognosis and limited therapeutic options. Alternating electrical fields with low intensity called “Tumor Treating Fields” (TTFields) are a new, non-invasive approach with almost no side effects and phase 3 trials are ongoing in advanced PDAC. We evaluated TTFields in combination with mild hyperthermia. Three established human PDAC cell lines and an immortalized pancreatic duct cell line were treated with TTFields and hyperthermia at 38.5°C, followed by microscopy, assays for MTT, migration, colony and sphere formation, RT-qPCR, FACS, Western blot, microarray and bioinformatics, and *in silico* analysis using the online databases *GSEA, KEGG*, Cytoscape-String, and Kaplan-Meier Plotter. Whereas TTFields and hyperthermia alone had weak effects, their combination strongly inhibited the viability of malignant, but not those of nonmalignant cells. Progression features and the cell cycle were impaired, and autophagy was induced. The identified target genes were key players in autophagy, the cell cycle and DNA repair. The expression profiles of part of these target genes were significantly involved in the survival of PDAC patients. In conclusion, the combination of TTFields with mild hyperthermia results in greater efficacy without increased toxicity and could be easily clinically approved as supporting therapy.

## Introduction

Pancreatic ductal adenocarcinoma (PDAC) has an exceptionally poor prognosis, high therapy resistance, and high rates of early metastasis (1). Currently, surgery remains the only chance to cure pancreatic cancer; however, >90% of patients relapse and die of their disease without additional treatment (2). For resectable tumors, surgery followed by adjuvant chemotherapy (gemcitabine plus capecitabine) is the standard of care. In the metastatic setting, FOLFIRINOX and nab-paclitaxel–gemcitabine are standard treatment options for patients with good performance status; both combinations have shown a survival advantage over the previous standard of gemcitabine monotherapy (2).

Tumor Treating Fields (TTFields) were first described in 2004 (3) and are low-intensity, intermediate-frequency, alternating electric fields delivered through noninvasive transducer arrays placed locoregionally around the anatomic region of the tumor (4). TTFields selectively target tumor cells or rapidly proliferating cells but not slowly proliferating tissues or cell cultures. The advantage of TTFields is that (1) the electrodes are noninvasive; (2) mainly cancer cells are targeted; (3) a thermal effect is not described due to the low intensity; and (4) nerves and muscles are not stimulated because of the high frequency of TTFields (3, 5, 6). Mechanistically, TTFields inhibit tumor cell division (7) by impairing the polymerization of microtubules and septin filaments during mitosis (7–9) and extending the duration of mitosis by the formation of defective mitosis structures (3, 7). This leads to aneuploidy and genomic instability, termed mitotic catastrophe, followed by cell death and senescence (7, 10).

Currently, the NovoTTF-100 or Optune system developed by Novocure Ltd. was approved by the FDA and EU for the treatment of therapy-refractory cancer entities such as glioblastoma and mesothelioma (11), and in Germany, a reimbursement mechanism has been in place since March 2020 (12). Phase 3 studies are currently underway for brain, lung, pancreas and uterine tumors, and a phase 2 study is being conducted for liver tumors. In addition, promising experimental data are available for numerous tumor entities (13), including the effects of TTFields on the PDAC cell lines AsPC-1 (14), CFPAC-I and HPAF-II (15). Upon treatment with TTFields at 150 kHz for 72 h, the cell number and viability were reduced, and the gemcitabine or radiation efficacy increased (14, 15). The PANOVA-2 trial recently demonstrated that the combination of TTFields and systemic chemotherapy is safe and tolerable in patients with advanced PDAC (16). Based on these results, the randomized PANOVA-3 trial is currently testing the efficacy of TTFields generated by the NovoTTF-100L(P) System in advanced PDAC in combination with palliative chemotherapy (17). However, TTFields remain controversial, e.g., in glioblastoma therapy, where the uptake of TTFields is increasing but remains limited (12).

Hyperthermia in oncology was first reported 5,000 years ago in a case of breast cancer (18). Today, the efficacy of locally applied, mild, moderate or severe hyperthermia, in the range from 39°C to 43°C was proven to be clinically effective in combination with radiotherapy and chemotherapy (19–21). Several applications at different temperatures are in clinical use, such as whole-body heating, or loco-regional hyperthermia. The latter also includes intraoperative superficial heating, hyperthermic intraperitoneal chemotherapy (HIPEC) and non-invasive deep regional heating (22). Moderate hyperthermia temperatures are used clinically in the form of whole-body hyperthermia e.g. in pain therapy. For this purpose, patients are heated for a duration of up to 1.5 h in insulated heat cabins. Alternatively, local application of mild hyperthermia in the tumor region can be achieved by infrared or microwave heating of the tissue. Randomized phase 2 and 3 clinical trials demonstrated that hyperthermia sensitizes tumors to cytotoxic therapy and thereby improved the outcome of cancers of the lung (23), breast (24), cervix (25), head and neck (26), skin (27), gastrointestinal tract (28), ovary (29), and sarcoma (30). For PDAC, the efficacy of hyperthermia has been demonstrated in the preclinical setting, e.g., in xenograft models in rats and mice, where the combination of gemcitabine and hyperthermia was more effective than single therapies (31). Mechanistically, hyperthermia alters the blood flow and nutrient distribution in tumors, induces a heat shock response followed by cell death in cancer cells (32), causes DNA damage (33), inhibits DNA repair (34), and induces cell cycle arrest (33, 35). However, the effective delivery of hyperthermia is often limited, and moderate to severe hyperthermia can have complications (36).

Our present study investigated whether a combination of mild hyperthermia of 38.5°C and TTFields of less than 1 V/cm would be more effective than each single treatment alone. We found a synergistic effect in PDAC cancer cells, whereas nonmalignant cells were not affected, and we identified the underlying signaling chains.

## Results

### Hyperthermia Enhances TTField-Mediated Inhibition of Viability

**Figure 1:**
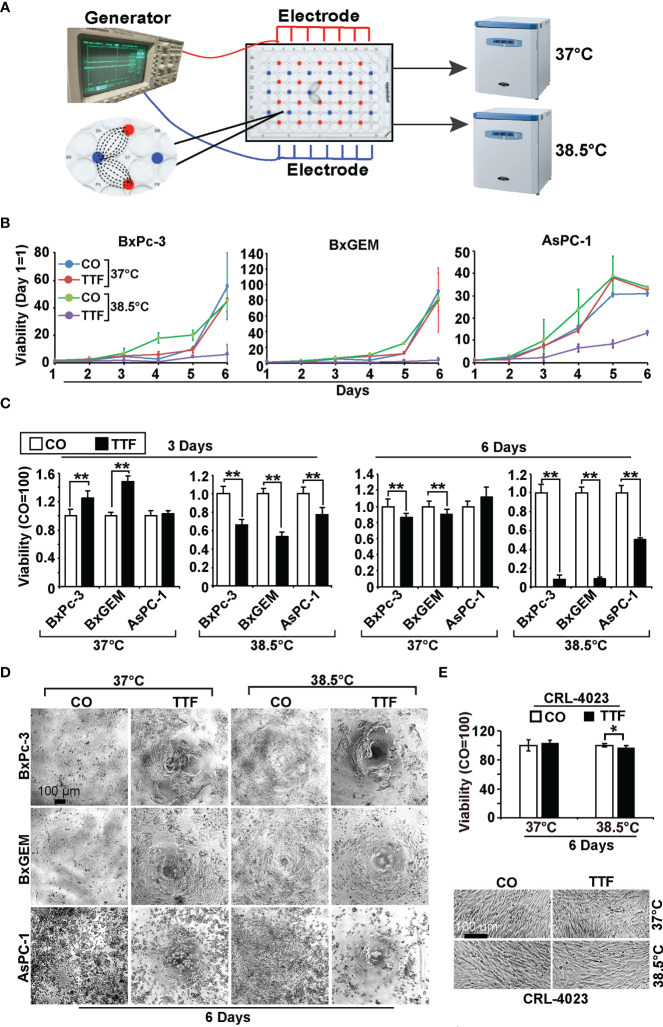
TTField-mediated inhibition of viability is enhanced by hyperthermia. **(A)** The scheme illustrates the treatment of PDAC cells with TTFields at a regular incubation temperature of 37°C or mild hyperthermia at 38.5°C. **(B)** The human PDAC cell lines AsPC-1, BxPC-3 and BxGEM were incubated at 37°C or 38.5°C in the presence (TTF) or absence (CO) of TTFields at 150 kHz and an intensity of less than 0.7 V/cm. To measure TTField effects as a function of treatment duration, PDAC cells were treated for 1 to 6 days in separate experiments. The examination of all 6 days together in one large experiment was not possible because the availability of the cell culture plates with antenna wires was limited. At the end of each single experiment, the viability was measured by MTT assay, the results were accumulated as a total curve for each cell line, and the values of each curve were normalized to start at 0 on day 1. **(C)** The cells were treated as described above, and the viability was analyzed 3 or 6 days later by MTT assay. The controls were set to 1. **(D)** The morphology of the cells, which were treated as described above, was microscopically examined at 100× magnification 6 days after treatment, and representative images are shown. The scale bar indicates 100 µm. **(E)** Likewise, the viability of the human, nonmalignant pancreatic duct cell line CRL-4023 was evaluated by MTT assay and microscopic evaluation at 100× magnification. Representative images of each treatment group of CRL-4023 cells 6 days after treatment are shown. Three independent experiments were performed at least in triplicate, and the data are presented as the means ± SD; *P < 0.05, **P < 0.01.

To examine the effect of TTFields on the viability of PDAC cell lines, we cultured BxPc-3, BxGEM and AsPC-1 cells in specially manufactured 96-well culture plates with electrodes to induce TTFields at frequencies of 150 kHz by a generator (**Figure 1A**). The cells in these culture plates were incubated at 37°C or 38.5°C for up to six days. The plates were then evaluated daily by MTT assay, which measures the cellular metabolic activity as an indicator of cell viability, proliferation and cytotoxicity. The values of each group were set to 0 at the start of the experiment at day 1. Whereas the MTT signal of cells cultured at 37°C in the presence or absence of TTFields continuously increased, it was even higher when the cells were cultured at 38.5°C (**Figure 1B**). A pronounced inhibition of viability was only seen upon the combination of hyperthermia and TTFields. These results became even clearer upon setting the controls to 1 and presenting the data in bar charts. While TTFields tended to increase the viability at 37°C, they significantly decreased it at 38.5°C after 3 days (**Figure 1C**). After 6 days, however, TTFields largely decreased the viability even at 37°C and almost completely at 38.5°C. Representative images of the cell morphology under microscopic magnification confirm the above results (**Figure 1D**). Interestingly, mild hyperthermia paired with TTFields failed to substantially inhibit the viability of the nonmalignant pancreatic ductal cell line CRL-4023, even 6 days after treatment (**Figure 1E**).

### Hyperthermia Increases TTField-Mediated Inhibition of Cancer Progression

**Figure 2:**
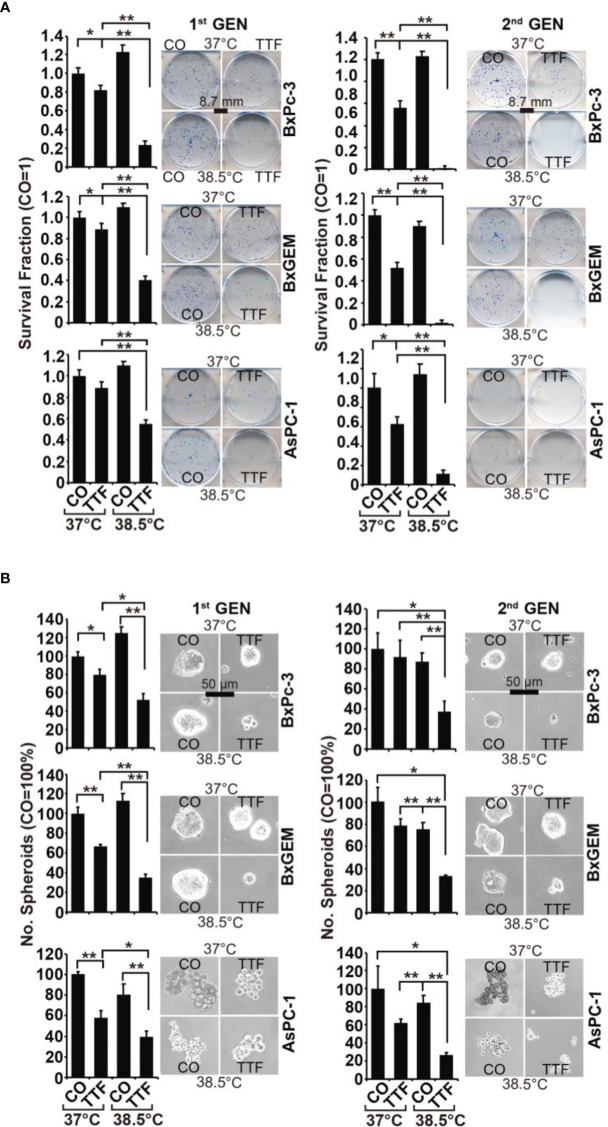
TTField-mediated inhibition of cancer stem cell features is enhanced by hyperthermia. **(A)** The cells were treated as described in **Figures 1A, B**, **(B)** After 3 days, the cells were detached from the cell culture plates by trypsinization and reseeded at clonal density (AsPC-1: 1,500 cells/well; BxPC-3 and Bx-GEM: 1,000 cells/well) in 6-well plates. The cells were cultured under regular conditions at 37°C without a medium change for 2 weeks, resulting in first-generation colonies (1^st^ GEN). The number of colonies was evaluated by fixing and Coomassie staining, followed by counting colonies with at least 50 cells using a dissecting microscope. The survival fraction and representative images are shown on the left. For the formation of second-generation (2^nd^ GEN) colonies, surviving cells from each group of first-generation colonies were collected, reseeded and analyzed as described above. **(B)** After treatment, as described in **Figure 1A**, the cells were seeded at a clonal density of 500 cells/well in ultralow-attachment 24-well plates in cell growth factor-supplemented serum-free culture medium to induce spheroid formation. Six days later, the first generation of spheroids developed, and the percentage of viable spheroids was evaluated by microscopy at 100× magnification and counting. Representative photographs and the means are shown on the left. For the formation of second-generation spheroids (2^nd^ GEN), surviving cells were collected from each group of first-generation spheroids and reseeded and analyzed as described above. The data are presented as the means ± SDs. *P < 0.05, **P < 0.01.

**Figure 3:**
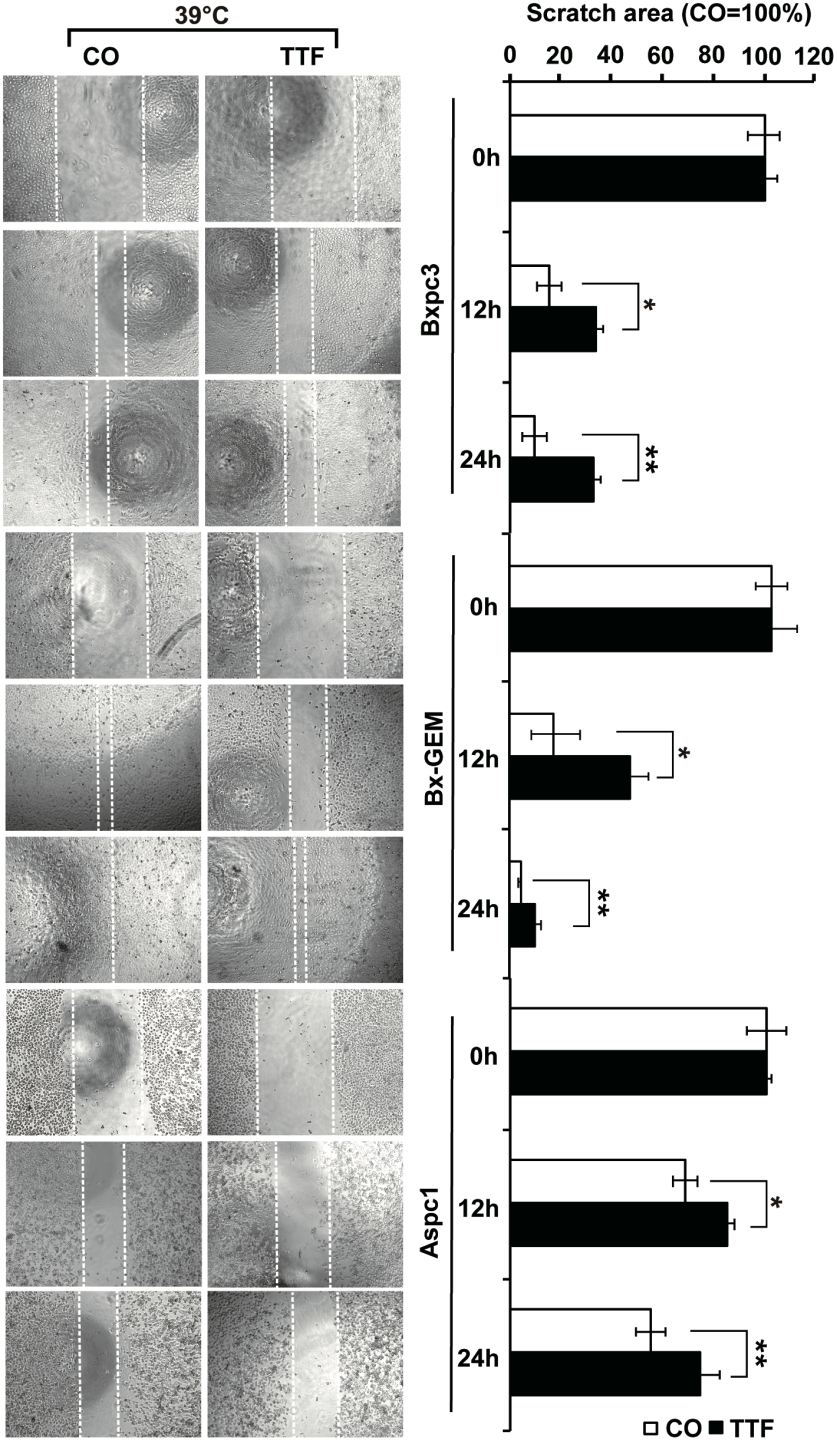
TTField-mediated inhibition of migration is stronger upon combination with hyperthermia. AsPC-1, BxPC-3 and Bx-GEM cells were seeded at a density of 4,000 cells/well in 96-well plates and grown to 90% confluency overnight. The cell layer was scratched with the tip of a 100-µL pipet, followed by treatment of the cells with TTFields at 38.5°C for 24 h. The controls (CO) were incubated at 38.5°C without exposure to TTFields. The closure of the wounded region was evaluated by microscopy at 0, 12 and 24 h after scratching. The gap width was measured using ImageJ. Representative images are shown on the left, and the means are shown in bar graphs on the right. The data are presented as the means ± SD. *P < 0.05, **P < 0.01.

To obtain information on the influence of mild hyperthermia paired with TTFields on stem cell properties, we studied the ability to form colonies. BxPc-3, BxGEM and AsPC-1 cells were treated with TTFields at 37°C or 38.5°C for 72 h. Then, the cells were reseeded at clonal density in 6-well plates, and colony formation was evaluated 14 days later. Whereas TTFields at 37°C only marginally reduced colony formation, they strongly reduced it at 38.5°C (**Figure 2A**). In contrast, hyperthermia alone significantly increased colony formation. For examination of the long-term effect, the surviving cells of each group were collected and equal cell numbers were reseeded at clonal density without further treatment. The resulting, so-called “second-generation” colonies were analyzed 14 days later. TTFields at 37°C reduced colony formation to approximately 50% to 60%, and TTFields in the presence of 38.5°C nearly completely abolished colony formation. Additionally, hyperthermia alone did not enhance colony formation, as observed in the short-term treatment. Similar results were obtained when the cells were treated and grown as spheroids (**Figure 2B**). Under these conditions, the effects were more pronounced upon reseeding the cells for second-generation spheroid formation. Next, we examined the effect of TTFields combined with mild hyperthermia or of mild hypothermia alone on migration and performed scratch assays. The closure of the wounded region was examined 12 h and 24 h later. The gap was significantly larger upon combination of TTFields and 38.5°C compared to 38.5°C alone (**Figure 3**). In contrast, the same experiments at 37°C were largely ineffective (data not shown). This result suggests again that the combination therapy also inhibits migration more than each single treatment option alone.

### Hyperthermia Increases TTField-Induced Differential Gene Expression

**Figure 4:**
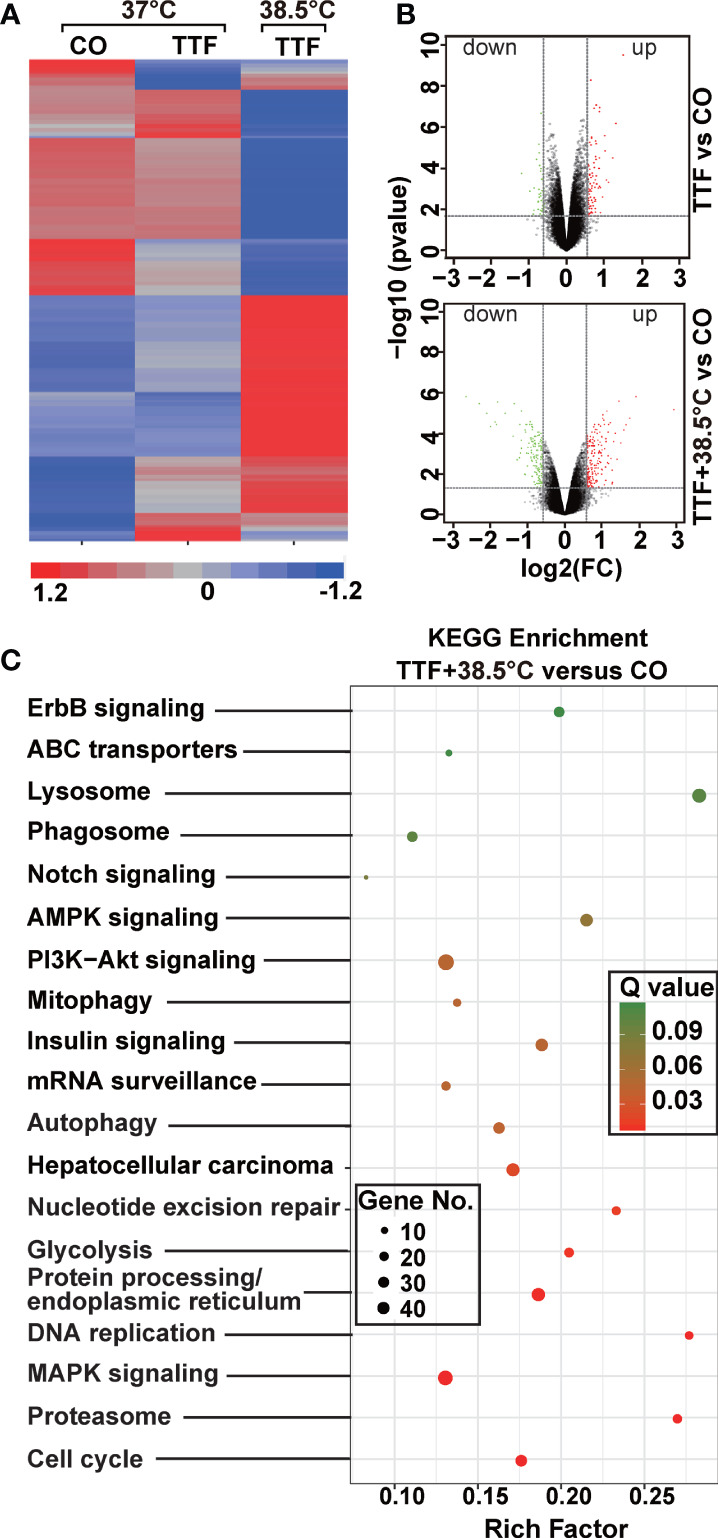
Gene array analysis demonstrates that hyperthemia enhances TTField-induced differential gene expression. **(A)** BxGEM cells were cultivated at 37°C in the absence (CO) or presence of TTFields (TTF) or were cultured at 38.5°C in the presence of TTFields (TTF+38.5°C) for 72 hours. Then, the mRNA was isolated, and gene array analysis was performed, followed by analysis of differentially regulated genes with a fold change of at least 1.5 and a significance of P < 0.05. Hierarchical cluster analysis was performed to organize the genes into clusters based on their expression similarities. Red: gene upregulation; Lilac: gene downregulation within a scale of 1.2 to -1.2. **(B)** Volcano plots were created and show the distribution of differentially expressed mRNAs in BxPc-3 cells cultured at 37°C in the absence (CO) or presence of TTFields (TTF *vs* CO) or at 38.5°C in the presence of TTFields (TTF+38.5°C *vs* CO). Red: (upregulated genes); Green (downregulated genes). The X-axis represents the expression profiles of multiple genes. The Y-axis represents the magnitude of gene expression changes. FC = 1.5, P < 0.05. **(C)** A KEGG enrichment analysis was performed to compare differentially regulated pathways between cells treated with TTFields + 38.5°C and control cells, which were cultivated at 37°C. The X-axis represents the rich factor, and the Y‐axis represents the KEGG terms. Rich factor: ratio of differentially expressed gene numbers annotated in this pathway term to all gene numbers annotated in this pathway term. The greater the Rich factor, the greater the degree of pathway enrichment. The size of the circles increases with the gene count. Different circle colors represent distinct Q values as indicated.

**Figure 5:**
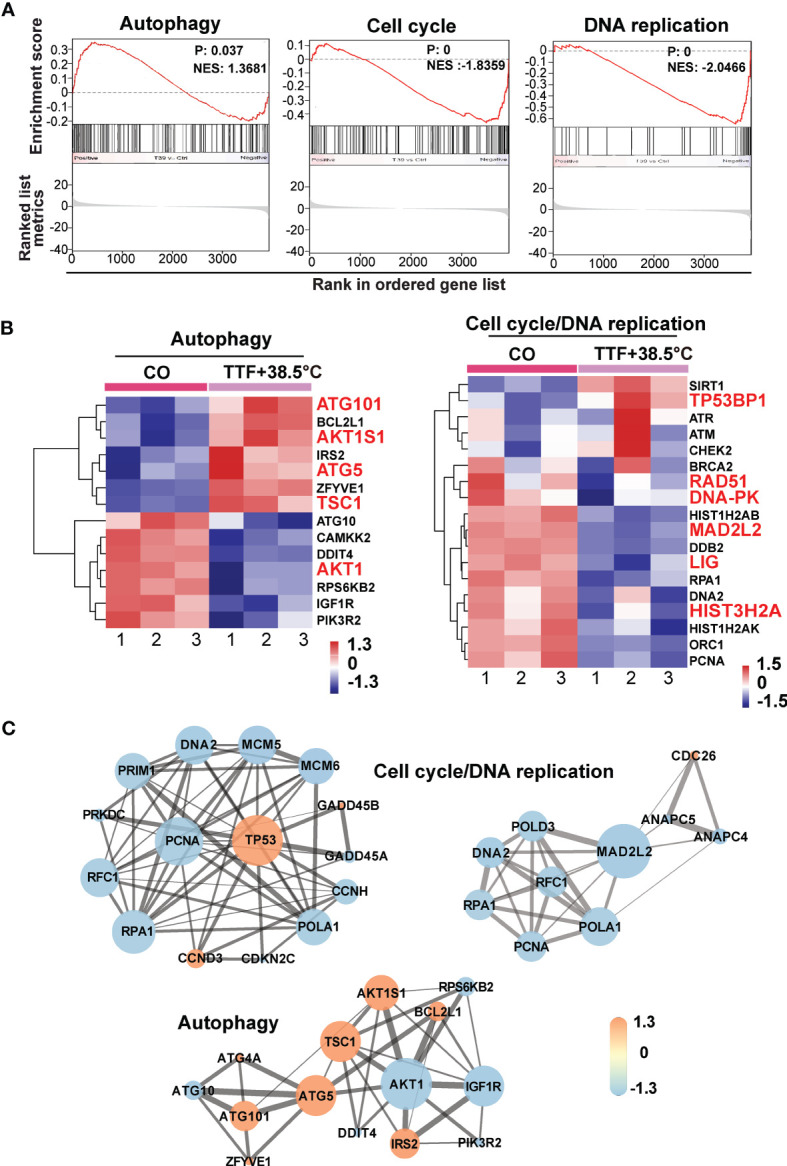
GSEA suggests TTField plus hyperthermia-mediated regulation of autophagy, the cell cycle and DNA replication. **(A)** The biological pathways that are enriched in the gene array lists were identified by gene set enrichment analysis (GSEA). Differentially regulated genes in BxGEM cells cultivated at 37°C or at 38.5°C plus TTFields were evaluated. In this way, clustering of an autophagy-related gene set, which positively correlated with the treatment group, was detected (Normalized enrichment score (NES) = 1.3681, P = 0.037, False discovery rate (FDR) = 0.13). Likewise, the clustering of a cell cycle-related gene set was found to be negatively correlated with the treatment group (NES = -1.8359, p = 0, FDR = 0.005). Additionally, the clustering of a DNA replication-related gene set was found to be negatively correlated with the treatment group (NES = -2.0466, P = 0, FDR = 0.006). **(B)** The most relevant genes of each gene set are shown as heat maps. The name of genes, which were identified by a Cytoscape/String analysis in the following are enlarged and marked in red. **(C)** The mRNA array data were analyzed with the open source software platform Cytoscape, which resulted in the visualization of networks of co-expressed genes related to the cell cycle, DNA replication and autophagy. The sizes of the circles indicate the interaction degrees of proteins. The circle colors indicate different fold changes. Orange: upregulation, Lilac: downregulation within a scale from -1.3 to 1.3 as indicated.

To further highlight gene expression changes, we treated BxGEM cells at 37°C and 38.5°C and used untreated cells cultured at 37°C as control. Seventy-two hours later, the RNA was harvested and a gene array analysis was performed. A heat map was generated and demonstrated well-separated gene clusters between the groups (**Figure 4A**). This finding was further highlighted by a volcano plot analysis of the array results, which exhibited a greater spreading of differentially regulated genes when TTFields plus were combined with 38.5°C compared to TTFields alone at 37°C (**Figure 4B**). Then, a KEGG enrichment analysis was performed, which led to the identification of 20 significantly up- or downregulated pathways closely related to tumorigenesis (**Figure 4C** and **Table 1**). Among the differentially regulated pathways were the ubiquitin proteasome system and autophagy, DNA repair, DNA replication, MAPK signaling and cell cycle regulation. Next, a GSEA analysis was performed and resulted in enrichment plots of autophagy, cell cycle and DNA replication (**Figure 5A**). The most significantly differentially regulated genes belonging to these pathways are shown in heat maps (**Figure 5B**). To identify the related signaling networks, the open source software platforms Cytoscape and String were utilized, which showed Tp53, PNCA, and MAD2L2 in the center of two cell cycle/DNA replication signaling networks and AKT1 in the center of an autophagy-related signaling network (**Figure 5C**). Thus, there is an interaction of the identified differentially regulated genes in biological signaling networks.

**Table 1:** GSEA analysis based on KEGG biological processes.

Name	NES	P	FDR
hsa03040_Spliceosome	1.73	0.00095	0.01413
hsa04141_Protein_processing_in_endoplasmic_reticulum	1.73	0.00037	0.00801
hsa04137_Mitophagy	1.66	0.00680	0.04629
hsa04010_MAPK_signaling_pathway	1.64	0.00018	0.00599
hsa03015_mRNA_surveillance_pathway	1.62	0.00710	0.04629
hsa04330_Notch_signaling_pathway	1.57	0.01913	0.08623
hsa04012_ErbB_signaling_pathway	1.43	0.03195	0.11690
hsa04142_Lysosome	1.41	0.02714	0.10552
hsa04140_Autophagy	1.37	0.03673	0.12996
hsa04151_PI3K-Akt_signaling_pathway	1.35	0.00767	0.04629
hsa04152_AMPK_signaling_pathway	-1.47	0.01406	0.07054
hsa04910_Insulin_signaling_pathway	-1.48	0.00741	0.04629
hsa02010_ABC_transporters	-1.5	0.03101	0.11556
hsa05225_Hepatocellular_carcinoma	-1.56	0.00233	0.02202
hsa00010_Glycolysis:–Gluconeogenesis	-1.82	0.00061	0.01010
hsa04110_Cell_cycle	-1.84	0.00020	0.00599
hsa03420_Nucleotide_excision_repair	-1.87	0.00120	0.01445
hsa03030_DNA_replication	-2.05	0.00020	0.00599
hsa03050_Proteasome	-2.21	0.00020	0.00599

The TTF+38.5°C group was compared with the control group (P < 0.05, FDR < 0.15).

### Hyperthermia and TTFields Enhance Autophagy and Cell Cycle Signaling

**Figure 6:**
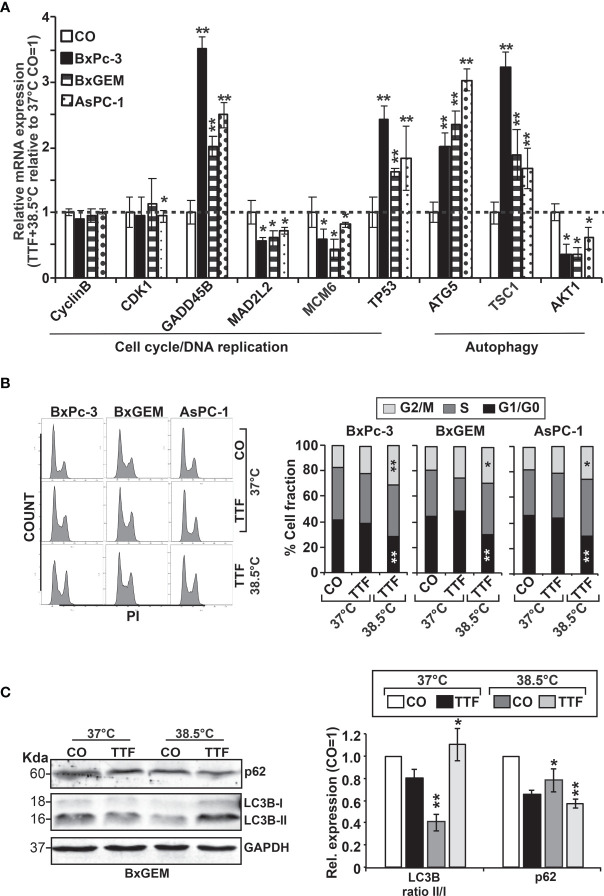
Functional studies confirm TTField plus hyperthermia-mediated autophagy, the cell cycle and DNA signaling. **(A)** BxPc-3, BxGEM and AsPC-1 cells were cultivated at 38.5°C plus TTFields or at 37°C in the controls, followed by isolation of mRNA three days later and RT-qPCR analysis. The gene expression level of each gene was normalized to that of *GAPDH*, and the relative mRNA expression of hyperthermia/TTFields compared to control cells is shown. The primer sequences are given in **Supplementary Table S1**, and a comparison of the expression levels is provided in **Table 1**. The results are presented as relative gene expression. For calculation, the 2^−ΔΔCt^ method of relative quantification given in the equation was used. **(B)** PDAC cells were seeded in 96-well plates and cultivated at 37°C in the absence (CO) or presence of TTFields (TTF) or were cultivated at 38.5°C plus TTFields (TTF+38.5°C) for 72 h. Then, the progression of the cell cycle was analyzed by staining the cells with propidium iodide, followed by flow cytometry. The results are shown as flow cytometry profiles (left) and cell cycle quantification in bar graphs (right). **(C)** The cells were cultivated at 37°C in the absence (CO) or presence of TTFields (TTF) or at 38.5°C plus TTFields, as indicated, and 72 h later, the proteins were isolated, and the expression of p62 and LC3 was examined by the use of specific antibodies and Western blot analysis. GAPDH served as the control for equal conditions. Three independent experiments were performed at least in triplicate, and the data are presented as the means ± SD; *P < 0.05, **P < 0.01.

For validation of our previous results, BxPc-3, BxGEM and AsPC-1 cells were treated with TTFields plus hyperthermia at 38.5°C, whereas cells cultured at 37°C without further treatment served as control. Seventy-two hours later, RT-qPCR was performed to examine the expression of the before identified target genes CyclinB, CDK1, GADD45B, MAD2L2, MCM6, TP53, ATG5, TSC1 and AKT1. The expression of each gene was normalized to the expression of GAPDH, and the gene expression of the TTField plus 38.5°C group was evaluated relative to the 37°C control, which was set to 1. GADD45B, TP53, ATG5 and TSC1 were significantly upregulated in all cell lines, whereas MAD2L2, MCM6 and AKT1 were significantly downregulated (**Figure 6A**). In contrast, the expression levels of CyclinB and CDK1 did not appreciably change. Most importantly, the expression patterns largely confirmed the expression data from the microarray assay, which is underlined by comparison of the results of both assays (**Table 2**).

**Table 2:** Comparison of RT-qPCR and microarray results.

Gene	Name of Gene	Microarray		qPCR		
		Fold change	FDR	2-△△CT		*P value*
*CCNB1*	CyclinB1	-1.07	7.26E-1	0.93		7.20E-1
*CDK1*	cyclin dependent kinase 1	-1.10	4.59E-3	1.12		8.10E-1
*GADD45B*	growth arrest and DNA damage inducible beta	1.36	4.03E-2	2.49		2.33E-3
*MAD2L2*	mitotic arrest deficient 2 like 2	-1.27	3.99E-3	0.61		2.16E-2
*MCM6*	minichromosome maintenance complex component 6	-1.50	7.85E-4	0.44		2.02E-2
*TP53*	tumour protein p53	1.23	1.06E-1	1.62		1.54E-3
*ATG5*	autophagy related 5	1.27	2.89E-2	2.32		3.43E-3
*TSC1*	TSC complex subunit 1	1.28	1.17E-2	1.88		7.99E-3
*AKT1*	AKT serine/threonine kinase 1	-1.3	2.09E-2	0.36		2.52E-3

To investigate the effect of TTFields on the cell cycle, the cells were cultured at 37°C or 38.5°C in the presence or absence of TTFields. Seventy-two hours later, the cells were stained with propidium iodide and examined by flow cytometry. TTFields combined with mild hyperthermia led to an increase of the G2/M phase, and a decrease of the G1 phase, whereas a statistically significant S phase arrest was not observed (**Figure 6B**).

To examine the influence induction of autophagy, the cells were cultured at 37°C or 38.5°C in absence or presence of TTFields. Seventy-two hours later, the expression of the autophagic flux indicator p62 and the presence of the autophagy marker LC3-II were examined by Western blot analysis. Whereas p62 expression decreased upon TTField treatment at 37°C and 38.5°C, indicating autophagy signaling, LC3-II was only clearly visible after combining TTFields with mild hyperthermia, as shown by a representative Western blot, quantification by Image J and a diagram with the means and standard deviations; also, the crude Western blot images are provided (**Figure 6C** and **Supplementary Figure S1**, data not shown). These data indicate that autophagy signaling occurred and was strongest when TTFields were combined with mild hyperthermia.

### Our Candidate Genes Are of Prognostic Relevance in PDAC

**Figure 7:**
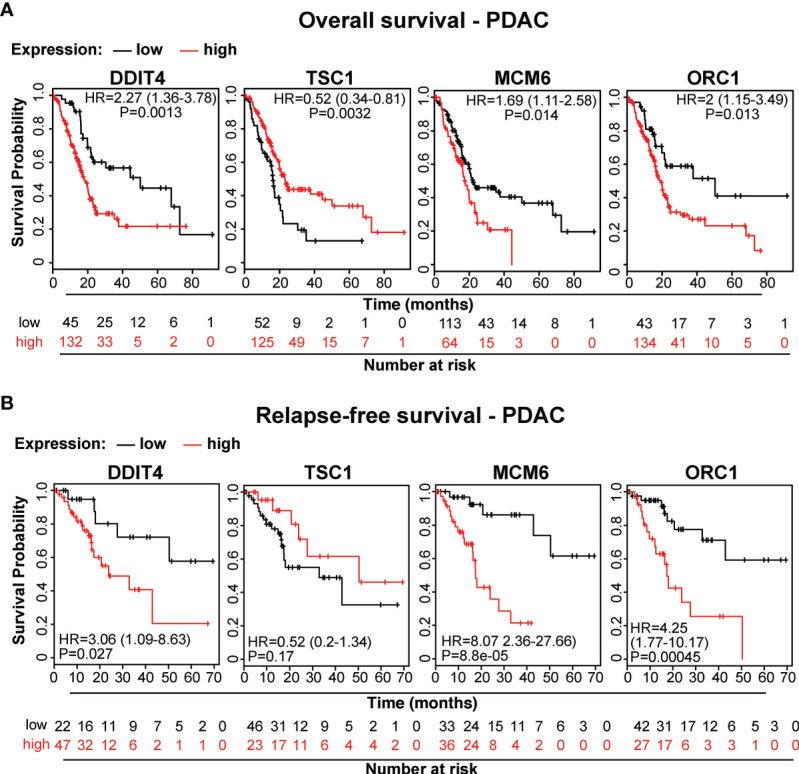
The expression levels of the candidate genes MCM6, ORC1, DITT4 and TSC1 correlate to overall and relapse-free survival in PDAC. **(A)** We searched the online database TCGA for existing data of the identified target genes and found PDAC-related expression and clinical data for *MCM6*, *ORC1*, *DITT4* and *TSC1.* Using these online data, we performed Kaplan-Meier analysis of overall survival and **(B)** relapse-free survival in relation to gene expression (data from TCGA-PAAD, pH 000178, N = 185). HR, Hazard ratio and the significance P are given.

To demonstrate the clinical relevance of candidate genes, which we identified by gene array analysis, we utilized TCGA public database and identified the availability of expression data paired with clinical data for DDIT4/REDD1, which is involved in cell growth, proliferation and survival; TSC1, which acts as a tumor suppressor; MCM6, which is involved in the cell cycle and DNA replication; and ORC1, which is involved in the initiation of DNA replication. Kaplan-Meier plots were created to visualize the relevance of expression of these genes in PDAC tissue and the related clinical outcome of patients (**Figures 7A, B**). Whereas high expression levels of DDIT4, MCM6 and ORC1 were related to significantly shorter survival, high expression of the tumor suppressor TSC1 was related to significantly longer survival, as expected. Together, these data confirm the relevance of the identified TTField plus hyperthermia-induced candidate genes identified by gene array analysis.

## Discussion

Here we investigated the therapeutic effects of TTFields at a frequency of 150 kHz, mild hyperthermia at 38.5°C, or the combination thereof. We deliberately chose a temperature below 39°C to avoid side effects as far as possible. We found that the combination treatment of PDAC cells nearly completely abolished the viability, metabolism, clonogenicity, migratory capacity, cell cycle progression and induced autophagy, while each single treatment was largely ineffective. In contrast, a nonmalignant pancreatic duct cell line was less affected, as we found by examination of the viability by MTT-assay. Although the latter finding does not exclude sub-lethal toxicity of TTFields combined with mild hypoxia to non-malignant cells, we assume that this is rather improbable. It is true that alternating electric TTFields affect the electric dipole moments of molecules in both, malignant and non-malignant cells. However, the field strength of the applied TTFields is quite low. Thus, a selective damage to malignant cells can be achieved by appropriate adjustment of the TTFields frequency to control the cell geometry-dependent amplification of the electric field in the division furrow. Due to the different geometry of malignant and non-malignant cells and a higher division rate of malignant cells, TTFields are suggested to interfere mainly with the mitotic spindle apparatus of malignant cells (7–9), followed by extension of the duration of mitosis by the formation of defective mitosis structures (3, 7), aneuploidy and genomic instability, and finally cell death and senescence (7, 10). Together, non-malignant or resting cells are less affected, because the selective effect of TTFields on cancer cells depends on cell size, doubling time and the optimal high frequency of TTFields (3, 5–7). These facts may exclude a severe damage of the vascular system as a key in order to keep the homeostasis of oxygen tension. Whether our therapeutic approach may target cancer-associated angiogenesis and thereby the endothelial cell as a checkpoint for immunological patrolling, as described (37), is a matter of debate and future evaluation.

We used the optimal TTField frequency of 150 kHz for the treatment of PDAC cells as described before by Giladi et al. (14). The combination with mild hyperthermia seems to be important because we demonstrated that the single treatments were less effective or even induced tumor growth upon short-term treatment. Thus, the viability of BxPc-3 and BxGEM cells was significantly increased 3 days after treatment with TTFields at 37°C, but inhibited after 6 days. Transferred to the treatment of patients with TTFields, which are delivered by a head hood or abdominal belt, these results suggest, that within the first days the tumor viability/metabolism may be stimulated, whereas later it is inhibited. Such a scenario of stimulation of cell proliferation by electric fields is e.g. described in the context of alternating fields with frequencies in the range between 60 kHz and 448 kHz, which excited nerve cells and heart muscle tissue by targeted depolarization of the membrane potential (38, 39). Likewise, improved fracture healing and increased bone growth seem to be stimulated by low-frequency alternating fields (40). These frequencies overlap with the frequency range of TTFields (41). We suspect that the field strength used by us also induced proliferation at the beginning of the treatment, whereas it may have induced perturbations in cell division at later time points and as described (7–9), and thereby inhibition of proliferation. The question is why we observed a stimulation of proliferation at an early time point in BxPc-3 and BxGEM cells, but not in AsPC-1 cells. This observation may be due to the 2-dimensiol growth of BxPc-3 and BxGEM cells and the 3-dimensional, spheroid-like growth of AsPC-1 cells and the accordant mitotic spindle orientation exposed to TTFields.

In our experimental setting, the treatment duration was a crucial factor because the observed therapeutic effects of single or combined TTFields plus mild hypothermia increased with time. Our experimental system is different from the Inovitro™ system provided by Novocure Ltd., which uses switching mechanisms to change the field direction to increase the efficiency (3). Patients are encouraged to wear the Novocure Ltd. device daily on the abdomen at the level of the pancreas to treat PDAC (17) or, in the case of glioblastoma, as a cap on the head (12). The TTFields that we induced were in one spatial direction, with no interaction with dividing cells oriented perpendicular to the electric field direction. Additionally, we were not able to use an optimal higher power due to the heat generated by the antenna wires; as a result, the electric field strengths in the middle of the wells were slightly less than 1 V/cm. Studies by Kirson et al. showed that starting at 1 V/cm with increasing electric field strength, the effect on mitotic cells increases during treatment (42). Recently, Ravin et al. developed a novel device to deliver TTFields to cell and tissue cultures, which solved the problem of unwanted heat production by continuous thermal regulation (43).

An important observation made in our study is that TTFields plus mild hyperthermia strongly inhibited the self-renewal potential by reducing the colony and spheroid formation abilities. Similar results were recently obtained in glioma cancer stem cell-like cells (44). In terms of the cancer stem cell hypothesis (45), these data suggest that the combination of TTFields with mild hyperthermia prevents tumor recurrence and therapy resistance, at least *in vitro*. However, the colonies and spheroids were not totally eliminated, suggesting that TTFields combined with mild hyperthermia should be rather seen as supporting approach in addition to standard cytotoxic therapy.

According to our gene array results, the combination of TTFields and mild hyperthermia inhibited pathways and signaling chains involved in DNA replication. We supported the gene array results by qRT-PCR analysis, Western blot analysis of candidate genes related to autophagy, FACS-analysis of cell cycle progression, as well as online database analysis including KEGG enrichment analysis, GSEA analysis, Cytoscape/String analysis, and Kaplan-Meier Plotter-based detection of the clinical relevance of the identified target genes for overall survival and relapse-free survival of patients suffering from pancreatic cancer.

Alterations in DNA replication have already been described for severe hyperthermia at a minimum temperature of 43°C, which damages DNA repair, increases DNA breaks, and ultimately converges into cell death (46–48). Although the temperature of 38.5°C tested here is far below the temperatures of severe hyperthermia, we observed an altered distribution of cells in the cell cycle and an accumulation of the G2/M population. Our results are similar to the findings of Giladi et al. (14) and Voloshin et al. (7, 49). Also, mild hyperthermia has increased the described spindle fiber damage known to be induced by TTFields (3, 7–9), which usually activates the spindle assembly checkpoint and induces cell cycle arrest in the G2/M-phase (50). This assumption is supported by our gene array results, where we found alterations in the expression levels of *MAD2L2*, *GADD45B*, *MCM5*, *MCM6* and *PRKDC*. All of these candidate genes indicate that the differential gene expression caused by TTFields in the presence of mild hyperthermia converges in the canonical pathways of cell cycle and DNA replication.

Moreover, we demonstrated that TTFields combined with mild hyperthermia induces autophagy in PDAC cells. This finding is consistent with published studies in glioblastoma cells (44, 51). However, autophagy is a double-edged sword and can induce cell death or prevent it, dependent on the cellular context, as we recently demonstrated in PDAC (52, 53). In the context of mild hyperthermia and TTFields, we found that the induction of autophagy supported cell death. Based on our gene array and *in silico* analysis, we speculate that the TTField-induced upregulation of autophagy is possibly dependent on the PIK3/AKT1 signaling pathway. This may be due to the cellular self-killing mechanism mediated by autophagy, or its protective effect in situations of cellular stress. Our data postulate that TTFields combined with mild hyperthermia induce DNA damage and delay DNA damage repair, which correlates with recent data showing that hyperthermia causes DNA damage (33) and/or interferes with DNA repair pathways (34). In this regard, hyperthermia has been reported to act as a radiosensitizer by interfering with DNA repair (54). Accordingly, we also observed alterations in important genes related to DNA repair, e.g., *53BP1*, *DNA-PK*, *MAD2L2*, *RAD51*, *H2A*, *LIG* and *DDB2*, upon combined treatment with TTFields and mild hyperthermia. We noted that the expression profiles of the identified candidate genes *MCM6*, *ORC1*, *DITT4* and *TSC1* directly correlated with the overall and recurrence-free survival of patients diagnosed with PDAC. These candidate genes may therefore be seen as novel progression markers in PDAC.

### Conclusion

We combined for the first time TTFields and mild hyperthermia of 38.5°C and demonstrate that this combination is more effective than each single treatment. Thus, the application of these two well-tolerated modalities may provide an efficient new treatment approach. A logical next step would now be the evaluation by *in vivo experiments* with mice, for which, however, the complex technical equipment would first have to be further developed. Then it would also have to be tested to apply the TTFields for quite short time in a repeated way with an interval time between repetitions of days, because such a protocol and its effects are interesting for the clinical application. As both individual treatments, TTFields and hyperthermia, are already in clinical use, their direct, combined application in patients, as co-treatment along with chemotherapy, would also be conceivable.

## Materials and Methods

### Cell Lines

The established human pancreatic cancer cell lines BxPc-3 and AsPC-1 and the human hTERT-HPNE immortalized nonmalignant pancreatic duct cell line CRL-4023 were obtained from the American Type Culture Collection (ATCC, Manassas, VA, USA) and cultured as described (55). Gemcitabine-resistant BxPc-3 cells (BxGEM) were selected from parental cells by continuous gemcitabine treatment with increasing concentrations up to 200 nM for more than one year (56). Negative mycoplasma cultures were confirmed by monthly mycoplasma tests. The cell lines were recently authenticated by a commercial service (DSMZ, Braunschweig, Germany).

### Generation of TTFields

Recently we developed in-house a device for applying TTFields to cells growing in 96-well flat-bottom plates and evaluated its functionality in control experiments (57). This TTField device enabled us to treat PDAC cells with an alternating voltage between two insulated wires. The antenna wires were arranged diametrically to each well within a 96-well plate and formed, together with a variable capacitor, the capacitive part of an electrical resonant circuit. The inductive part of the resonant circuit was the secondary coil of a transformer whose primary coil was connected to a function generator (HP 3310A, Hewlett Packard, USA) and a customized preamplifier. An alternating low-voltage signal with a fixed frequency between 100 and 300 kHz was generated in the primary coil, and the resonant circuit was tuned to resonance using the variable capacitor. At resonance, voltage amplitudes *U_eff_* of up to 500 V were generated in the resonant circuit. We calculated the maximum value of the electric field strength in the area of the cells under highly simplified conditions. The antenna wires ended at the bottoms of the 96-well plates on which the cells grew. Considering only the 2-dimensional case on the bottom plane of the 96-well plates, the antenna wires can be approximated as point-like charges, and Coulomb’s law in equation (1) was used for the calculation.


(1) $$\vec{E}(\vec{r})=\frac{Q}{4\pi \epsilon_{0}\epsilon_{r}}\frac{\vec{e}}{r^{2}}$$

In this equation, *Q* is the field-generating charge, $\vec{e}$ is the unit vector for the field direction, *ϵ*_0_ = 8.85 * 10^–12^ *As*/*Vm* is the vacuum permittivity, *ϵ_r_* is the relative permittivity and *r* is the distance to the charge. The total field is formed as a superposition of 2-point charges according to equation (1). The calculations were carried out only for the straight line connecting the two-point charges. The field effects of charges outside the calculation plane were ignored. With the applied voltage *U_eff_* = 500 V, we calculated the charge *Q* with equation (2):


(2) $$Q=CU_{eff}$$

with the capacitance *C* of the antenna wires.

The total capacitance *C* was calculated according to equation (3), taking into account, that there were different dielectric materials between the antenna wires: air, plastic, and the saline cell medium.


(3) $$C={\left(\frac{2}{C_{air}}+\frac{2}{C_{p}}+\frac{1}{C_{Med}}\right)}^{-1}$$

*C_air_*, *C_P_*, and *C_Med_* were calculated with equation (4):


(4) $$C_{k}=\epsilon_{k}\epsilon_{0}\frac{A}{d_{k}}$$

with *k* = *air*, *P*, *Med* and *A* = *πd_w_L_w_*/2, *d_w_* = 0.5*mm*, *L_w_* = 10*mm*.

*C_air_* is the capacitance of the air gap between the antenna wire and the adjacent well wall using *ϵ_air_* = 1 and *d_air_* = 3 mm, *C_P_* is the capacitance of the plastic well wall using *ϵ_p_* = 2 and *d_P_* = 1 mm, and *C_Med_* is the capacitance of the cell medium using *ϵ_Med_* = 75 and *d_Med_* = 5 mm. Depolarization factors due to the geometry of the various dielectrics were not taken into account.

For treatment of cell lines with TTFields, the cells were seeded in 96-well plates at a density of 2000-4000 cells/ml in 200 µl of medium per well. After installation of the antenna wires for the treatment with TTFields, the plates were placed in the incubator. The cell culture medium was not changed during treatment.

### Examination of Viability/Metabolic Activity by MTT Assay

PDAC cell lines were resuspended at a concentration of 2 to 4x 10^3^ cells/ml, and 200 µl per well of a 96-well microplate were seeded. We minimized the counting errors by continuous shaking of the cell suspension and the use of a multi-head pipette for cell seeding. Also, the different cell concentrations from 2 to 4x 10^3^ cells/ml per batch enabled us to took those for evaluation in which the control group grew to nearly 100% density after 6 days. After treatment, 20 µL of the 5 mg/mL yellow MTT tetrazolium salt (3-(4,5-dimethylthiazol-2-yl)-2,5-diphenyltetrazolium bromide) were added to each well. The plates were incubated for 3.5 h at 37°C. The principle of this assay is that viable, metabolically active cells, but not dead or dying cells, contain NAD(P)H-dependent oxidoreductase enzymes, which reduce the yellow MTT to purple formazan crystals. By microscopic inspection, we detected the formation of formazan crystals and subsequently the medium was carefully removed, 200 µL DMSO was added and the cells were agitated on a shaker for 10 min at 37°C until the formazan crystals were completely dissolved. Finally, the optical density of the purple colour, which reflects mitochondrial activity, was measured at 570 nm by the use of an ELISA reader.

### Western Blot Analysis

Proteins were harvested by the use of RIPA Lysis Buffer and the protein concentration was determined by the use of the BCA Protein Assay Kit (both from Abcam, Cambridge, UK). After denaturation by boiling for 5 min, the samples were incubated on ice and then separated along with a commercial protein ladder by SDS-PAGE. A semi-dry system was used for the transfer of separated proteins in the gel to a PVDF membrane. The membrane was blocked by shaking in 3% BSA solution, followed by incubation with primary antibodies and thereafter with IRDye® infrared.

Dye-conjugated secondary antibodies (LI-COR Biosciences GmbH, Bad Homburg, Germany). The infrared intensity was measured by the use of an Odyssey CLx Infrared Imaging System (LI-COR Biosciences GmbH). The primary antibodies were rabbit polyclonal anti-LC3B and anti-P62 (Abcam, Cambridge, UK) and rabbit monoclonal anti-GAPDH antibody (Abcam).

### Cell Cycle Analysis

Seventy-two hours after treatment with TTFields at 37°C or 38.5°C as described above, the cells were harvested, washed and 5 ml ice-cold 70% EtOH was added drop-by-drop while vortexing. Subsequently, the samples were incubated at -20°C overnight. After PBS-washing, the cells were resuspended in 0.5 mL of the PI/RNase Staining Buffer (Becton Dickinson, Heidelberg, Germany) and stored for 15 min at room temperature. The cell cycle was analyzed by flow cytometry and the use of a FACSCalibur™ device (Becton Dickinson). The measured data were assessed with FLOWJO software (FLOWJO, LLC, Ashland, USA) to determine the cell fractions in the G1, S, and G2+M phases of the viable cell population.

### Colony-Forming Assay

Seventy-two hours after treatment with TTFields at 37°C or 38.5°C as described above, the cells were detached by trypsinization and reseeded at low density in 6-well plates (BxPc-3 and BxGEM, 1000 cells/well; AsPC-1, 800 cells/well) in six-fold approaches. The cells were incubated in a 37°C incubator for 10-14 days without changing the cell culture medium until considerably appropriate colonies were observed under the microscope in plates containing untreated control cells. Subsequently, the cells were washed with 10 mL PBS and 2 mL of 3.7% paraformaldehyde (PFA), was added for 10 min at room temperature. The fixation solution was removed, and 2 mL of 70% EtOH were added for 10 min. Finally, the cells were stained with 0.05% Coomassie blue, followed by washing with water. The plates were dried overnight. Colonies consisting of a minimum of 50 cells were counted under a stereomicroscope. The percentage of plating efficiency was evaluated by normalising the values obtained for treated cells to the values of non-treated cells. After normalisation, the value of non-treated cultures was set to 1. For the analysis of the second generation of colony formation, living cells were harvested and re-plated at low density in 6-well plates.

### Spheroid Assay

Seventy-two hours after treatment with TTFields at 37°C or 38.5°C as described above, the cells were seeded at a density of 1x 10^3^ cells/mL in 500 µL NeuroCult™ NS-A basal serum-free medium supplemented with 20 ng/mL hEGF, 10 ng/mL bFGF and 2 μg/mL heparin (STEMCELL Technologies Cambridge, US) per well in 24-well Sphera Low-Attachment Surface plates (ThermoFisher Scientific, Waltham, MA, USA) for spheroid formation. The spheroids were photographed after 5 days, and cell spheroids were identified according to their typical shape and size and the percentage of spheroids was calculated. To evaluate secondary spheroid formation, equal numbers of surviving cells of the first round of spheroid-formation were reseeded. For quantification of the percentage of spheroid forming cells, the cells were seeded at one cell per well in 96-well plates. Wells with more than one cell were excluded from evaluation.

### Wound Healing/Scratch/Migration Assay

The cells (1x 10^3^/well) were seeded in 96-well plates and grown to >90% confluence overnight. The cell layer was scratched with the fine end of a 10 μL pipette tip (time 0). Then the cells were exposed to TTFields at 37°C or 38.5°C. The closure of the wounded region was evaluated 12 h and 24 h later. Pictures were taken by the use of a Nikon Eclipse TS 100-F inverted microscope equipped with a camera. The images and the closure of the gap were analyzed with the computer program Image J (58).

### Real-Time Quantitative PCR

Seventy-two hours after treatment with TTFields at 37°C or 38.5°C as described above, the cells were harvested and total RNA was isolated by the use of the RNeasy^®^ Mini Kit (Qiagen, Hilden, Germany) according to the manufacturer’s instructions. The concentration was determined by the use of a Nanodrop 2000 spectrophotometer (ThermoFisher Scientific, Waltham, MA, USA) and the RNA was stored at -80°C until use. A total of 100 ng mRNA were reverse transcribed using the High Capacity RNA to cDNA™ Kit according to the instructions of the manufacturer (ThermoFisher Scientific, Waltham, MA, USA). The cDNA was diluted in 200 µl RNAse-free water and 1 μL cDNA was used immediately as template for real-time PCR, which was performed with 1 μL of forward and reverse primers for the genes of interest and SSoAdvanced SYBR Green Supermix (Qiagen, Hilden, Germany). The primer sequences were created by the use of PrimerBank, available for free online (https://pga.mgh.harvard.edu/primerbank/), and the primer sequences for *MAD2L2*, *GADD45B*, *MCM6*, Cyclin B1 (*CCNB1*), *CDK1*, *ATG5*, *TSC1*, *TP53* and *AKT1* are shown in **Table 3**. The PCR conditions were 10 min 95°C as initial denaturation step, followed by 40 cycles of 15 s denaturation at 95°C, 1 min annealing at 60°C, followed by cooling down to 4°C. All reactions were run in duplicate. Melt curve analysis for each pair of primers and agarose gel electrophoresis of the PCR products ensured the specificity of the primers. The gene expression level of each target gene was normalised to that of *GAPDH*. The results are presented as relative expression value (REV) by using the 2^−ΔΔCt^ method of relative quantification given in equation (6).


(5) $$REV=2^{Ct\;value\;of\;GAPDH-Ct\;value\;of\;the\;gene\;of\;interest}$$

**Table 3:** Primer Sequences used for RT-qPCR.

Symbol	Sequence
*MAD2L2*	Fw 5′-CGAGTTCCTGGAGGTGGCTGTGCATC-3′
	Rev 5′- CTTGACGCAGTGCAGCGTGTCCTGGATA-3′
*GADD45B*	Fw 5′-ACGAGTCGGCCAAGTTGATG3′
	Rev 5′-GGATGAGCGTGAAGTGGATTT-3′
*MCM6*	Fw 5′- GAGGAACTGATTCGTCCTGAGA-3′
	Rev 5′- CAAGGCCCGACACAGGTAAG -3′
*CCNB1*	Fw 5′- AATAAGGCGAAGATCAACATGGC-3′
	Rev 5′- TTTGTTACCAATGTCCCCAAGAG -3′
*CDK1*	FW 5′- AAACTACAGGTCAAGTGGTAGCC-3′
	Rev 5′-TCCTGCATAAGCACATCCTGA -3′
*ATG5*	FW 5′- AAAGATGTGCTTCGAGATGTGT -3′
	Rev 5′- CACTTTGTCAGTTACCAACGTCA-3′
*TSC1*	FW 5′- CAACAAGCAAATGTCGGGGAG -3′
	Rev 5′- CATAGGGCCACGGTCAGAA 3′
*TP53*	FW 5′- CAGCACATGACGGAGGTTGT -3′
	Rev 5′- TCATCCAAATACTCCACACGC 3′
*AKT1*	FW 5′- ATGAACGACGTAGCCATTGTG-3′
	Rev 5′- TTGTAGCCAATAAAGGTGCCAT-3′
*GAPDH*	Fw 5′-GAAGGTGAAGGTCGGAGTC-3′
	Rev 5′-GAAGATGGTGATGGGATTTC-3′

By referring to each REV value of the target gene, the fold change (FC) can be calculated using equation (7).


(6) $$Fold\;change=\frac{REV\;treated\;cells}{REV\;untreated\;cells}$$

### mRNA Microarray Analysis

mRNA was isolated from untreated or TTField-treated BxGEM cells grown at 37°C or 38.5°C using the RNeasy Kit (QIAGEN, Hilden, Germany) according to the manufacturer’s instructions. Microarray analyses was performed at the Microarray-Analytic Center of the Medical Faculty Mannheim using Clariom™ D Assays (Thermo Fisher Scientific, Dreieich, Germany). Biotinylated antisense complementary DNA (cDNA) was prepared based on a standard Affymetrix labeling protocol with the GeneChip^®^ WT Plus Reagent Kit and the GeneChip^®^ Hybridization, Wash and Stain Kit. Thereafter, hybridization on the chip was performed in a GeneChip hybridization oven 640, staining was performed in the GeneChip Fluidics Station 450, and the chip was then scanned with a GeneChip Scanner 3000. The custom CDF version 22 with ENTREZ-based gene definitions was used to annotate the arrays (59). The raw fluorescence intensity values were normalized by applying quantile normalization and RMA background correction. One-way analysis of variance (ANOVA) was applied; a fold change of 1.5 was used for the selection of differentially expressed genes using commercial SAS JMP10 Genomics version 6 (SAS Institute, Cary, USA). A false positive rate/false discovery rate (FDR) <0.15 was considered to be the level of significance. The accession number of the gene array at ArrayExpress (https://www.ebi.ac.uk/arrayexpress/) is #E-MTAB-10270 (BxGEM, control, TTF, TTF+hyperthermia 38.5°C).

### *In Silico* Analysis of the mRNA Microarray Results

The gene array-derived list of differentially expressed genes was further correlated for their biological function and involvement in signaling pathways. For the selection of differentially expressed genes, an absolute value of the logarithmic fold change (log FC) ≥1.3 and a cutoff of P<0.05 were chosen to identify statistically significant pathways.

**Heat maps and volcano plots** were created with the free software environment for statistical computing and graphics R (https://www.R-project.org/).

**Gene set enrichment analysis (GSEA)** was performed using the *fgsea* package available in the open-source software Bioconductor (https://bioconductor.org/packages/release/bioc/html/fgsea.html).

The freely available online database resource **KEGG (Kyoto Encyclopedia of Genes and Genomes,** https://www.genome.jp/kegg/) was used for the selection of relevant biological functions and pathways with enrichment scores of P<0.05.

The open source platform **String 11.0** (https://string-db.org) was used to collect, score and integrate publicly available sources of protein–protein interaction data and to supplement it with calculations and predictions. By the use of the **Cytoscape StringApp (** http://apps.cytoscape.org/apps/stringapp), we visualized the identified protein-protein-interaction network based on the obtained differentially expressed candidate genes.

### Evaluation of Target Genes by TCGA and Kaplan-Meier Plotter Analysis

The Cancer Genome Atlas (TCGA) is a publicly available online database (https://www.cancer.gov/about-nci/organization/ccg/research/structural-genomics/tcga) with over 20,000 primary cancer and matched normal samples spanning 33 cancer types with matched molecular and clinicopathological data. TCGA was used for evaluation of the identified target genes DDIT4, TSC1, MCM6 and ORC1 by Kaplan-Meier analysis using the online available Kaplan-Meier Plotter (http://kmplot.com/analysis/index.php?p=service). mRNAs expression data of the above-mentioned target genes from human PDAC tumor tissue were available and used to analyze the overall survival (OS) and recurrence-free survival (RFS) of patients. The database divides patient samples into high expression groups and low expression groups according to the median values of mRNA expression and validates them by a Kaplan–Meier survival curve. Information on number of patients, median values of mRNA expression, 95% confidence interval (CI), hazard ratio (HR), and P-value can be found on the Kaplan–Meier Plotter web page (http://kmplot.com/analysis/index.php?p=service). A P-value <0.05 was considered as statistically significant. The log-rank test was used to calculate the statistical significance of the differences observed among the Kaplan-Meier curves.

### Statistical Evaluation

Statistical analyses were performed using JMP14.0.0 (SAS Institute, Cary, USA) or Prism 7.0 (GraphPad Software, San Diego, California, USA). For most of the experiments, Dunnett’s tests were used to calculate the P values; nontreated cells at 37°C served as the control group. For cell migration, Student’s t-tests of independent samples were used to calculate the P values. All experiments were repeated a minimum of three times, and the data are presented as the mean ± standard deviation (SD). The null hypothesis was rejected when P <0.05 (*P <0.05 and **P <0.01).

## Data Availability Statement

The datasets presented in this study can be found in online repositories. The names of the repository/repositories and accession number(s) can be found in the article/**Supplementary Material**.

## Ethics Statement

The studies involving human participants does not apply because we used anonymous patient data, which are available for free from online databases, such as The Cancer Genome Atlas Program. Written informed consent for participation was not required for this study in accordance with the national legislation and the institutional requirements.

## Author Contributions

LB, MS, IH: Concept and design. MS, LB; TP, LL, SZ: Development of methodology. LB, TP, CT, SZ: Acquisition of data. LB, MS, WG, CT, IH: Analysis and interpretation of data. LB, MS, IH: Writing, review and/or revision of the manuscript. All authors contributed to the article and approved the submitted version.

## Funding

This study was supported by grants to IH from the German Research Council (DFG HE 3186/15-1), Hanns A. Pielenz-Stiftung, Heidelberger Stiftung Chirurgie, Dietmar Hopp-Stiftung, and Klaus Tschira Stiftung. The authors declare that this study received funding from Karsten Burmeister/BIMAG Bau- und Industriemaschinen GmbH. The funder was not involved in the study design, collection, analysis, interpretation of data, the writing of this article or the decision to submit it for publication.

## Conflict of Interest

The authors declare that the research was conducted in the absence of any commercial or financial relationships that could be construed as a potential conflict of interest.

## Publisher’s Note

All claims expressed in this article are solely those of the authors and do not necessarily represent those of their affiliated organizations, or those of the publisher, the editors and the reviewers. Any product that may be evaluated in this article, or claim that may be made by its manufacturer, is not guaranteed or endorsed by the publisher.

## Abbreviations

ANOVA, One-way analysis of variance; CI, Confidence interval; HR, Hazard ratio; FDR, False discovery rate; GSEA, Gene set enrichment analysis; KEGG, Kyoto Encyclopedia of Genes and Genomes; MTT, 3-(4,5-dimethylthiazol-2-yl)-2,5-diphenyltetrazolium bromide; OS, Overall survival; RFS, Recurrence-free survival; SD, Standard deviation; PDAC, Pancreatic ductal adenocarcinoma; TCGA, The Cancer Genome Atlas; TTFs, Tumor Treating Fields.
