# Corrigendum: Establishment of Tumor Treating Fields Combined With Mild Hyperthermia as Novel Supporting Therapy for Pancreatic Cancer

**DOI:** 10.3389/fonc.2022.889215

**Published:** 2022-03-31

**Authors:** Liping Bai, Tobias Pfeifer, Wolfgang Gross, Carolina De La Torre, Shuyang Zhao, Li Liu, Michael Schaefer, Ingrid Herr

**Affiliations:** ^1^ Molecular OncoSurgery, Section Surgical Research, Department of General, Visceral and Transplantation Surgery, University of Heidelberg, Heidelberg, Germany; ^2^ Medical Research Center, Medical Faculty Mannheim, University of Heidelberg, Heidelberg, Germany; ^3^ Department of Hematology, Oncology and Rheumatology, Internal Medicine V, University Hospital of Heidelberg, Heidelberg, Germany

**Keywords:** pancreatic ductal adenocarcinoma, hyperthermia, tumor treating fields, alternative therapies, bioinformatics and computational biology

In the original article, there was a mistake in **Figure 2A** as published. The representative images of colony formation “AsPC-1/CO/38.5°C, 1^st^ generation” and “BxGEM/CO/38.5°C, 2^nd^ generation” were mixed up by mistake. The corrected **Figure 2** appears below.

**Figure 2:**
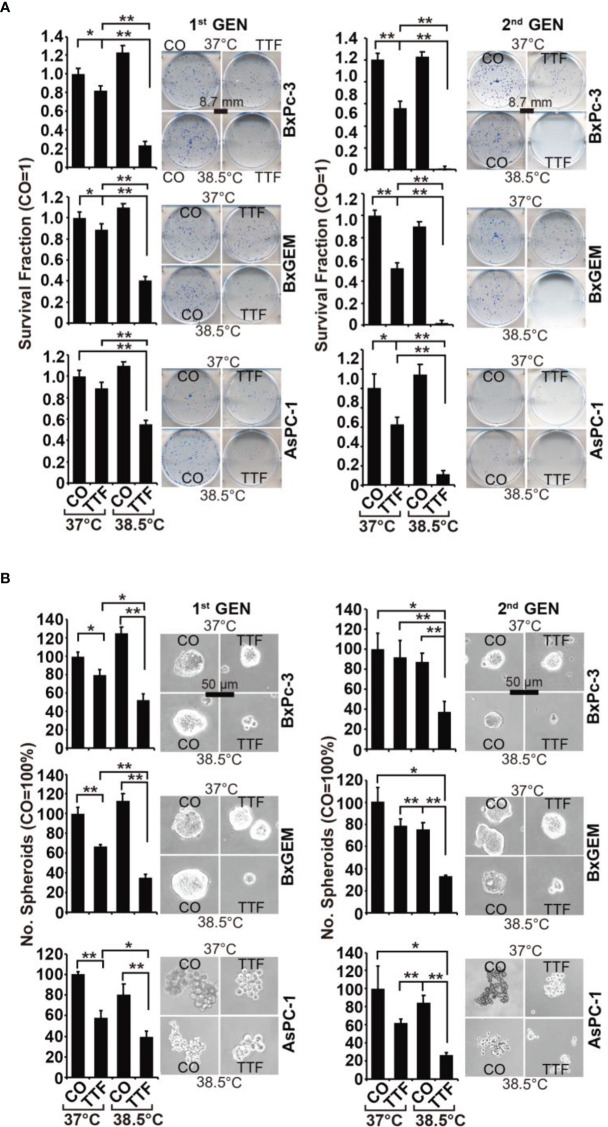
TTField-mediated inhibition of cancer stem cell features is enhanced by hyperthermia. **(A)** The cells were treated as described in Figures 1A, B, **(B)** After 3 days, the cells were detached from the cell culture plates by trypsinization and reseeded at clonal density (AsPC-1: 1,500 cells/well; BxPC-3 and Bx-GEM: 1,000 cells/well) in 6-well plates. The cells were cultured under regular conditions at 37°C without a medium change for 2 weeks, resulting in first-generation colonies (1st GEN). The number of colonies was evaluated by fixing and Coomassie staining, followed by counting colonies with at least 50 cells using a dissecting microscope. The survival fraction and representative images are shown on the left. For the formation of second-generation (2nd GEN) colonies, surviving cells from each group of firstgeneration colonies were collected, reseeded and analyzed as described above. **(B)** After treatment, as described in Figure 1A, the cells were seeded at a clonal density of 500 cells/well in ultralow-attachment 24-well plates in cell growth factor-supplemented serum-free culture medium to induce spheroid formation. Six dayslater, the first generation of spheroids developed, and the percentage of viable spheroids was evaluated by microscopy at 100× magnification and counting. Representative photographs and the means are shown on the left. For the formation of second-generation spheroids (2nd GEN), surviving cells were collected from each group of first-generation spheroids and reseeded and analyzed as described above. The data are presented as the means ± SDs. *P < 0.05, **P < 0.01.

The authors apologize for this error and state that this does not change the scientific conclusions of the article in any way.

## Publisher’s Note

All claims expressed in this article are solely those of the authors and do not necessarily represent those of their affiliated organizations, or those of the publisher, the editors and the reviewers. Any product that may be evaluated in this article, or claim that may be made by its manufacturer, is not guaranteed or endorsed by the publisher.

