# Corrigendum: Establishment of tumor treating fields combined with mild hyperthermia as novel supporting therapy for pancreatic cancer

**DOI:** 10.3389/fonc.2024.1343421

**Published:** 2024-02-29

**Authors:** Liping Bai, Tobias Pfeifer, Wolfgang Gross, Carolina De La Torre, Shuyang Zhao, Li Liu, Michael Schaefer, Ingrid Herr

**Affiliations:** ^1^ Molecular OncoSurgery, Section Surgical Research, Department of General, Visceral and Transplantation Surgery, University of Heidelberg, Heidelberg, Germany; ^2^ Medical Research Center, Medical Faculty Mannheim, University of Heidelberg, Heidelberg, Germany; ^3^ Department of Hematology, Oncology and Rheumatology, Internal Medicine V, University Hospital of Heidelberg, Heidelberg, Germany

**Keywords:** pancreatic ductal adenocarcinoma, hyperthermia, tumor treating fields, alternative therapies, bioinformatics and computational biology

**Error in** **Figure 3**

In the original article, there was a duplicated, representative image in ***Figure 3*** as published. The representative image of “AsPC-1/CO/12h” was unintentionally swapped. The revised and correct Figure appears below.

**Figure 3:**
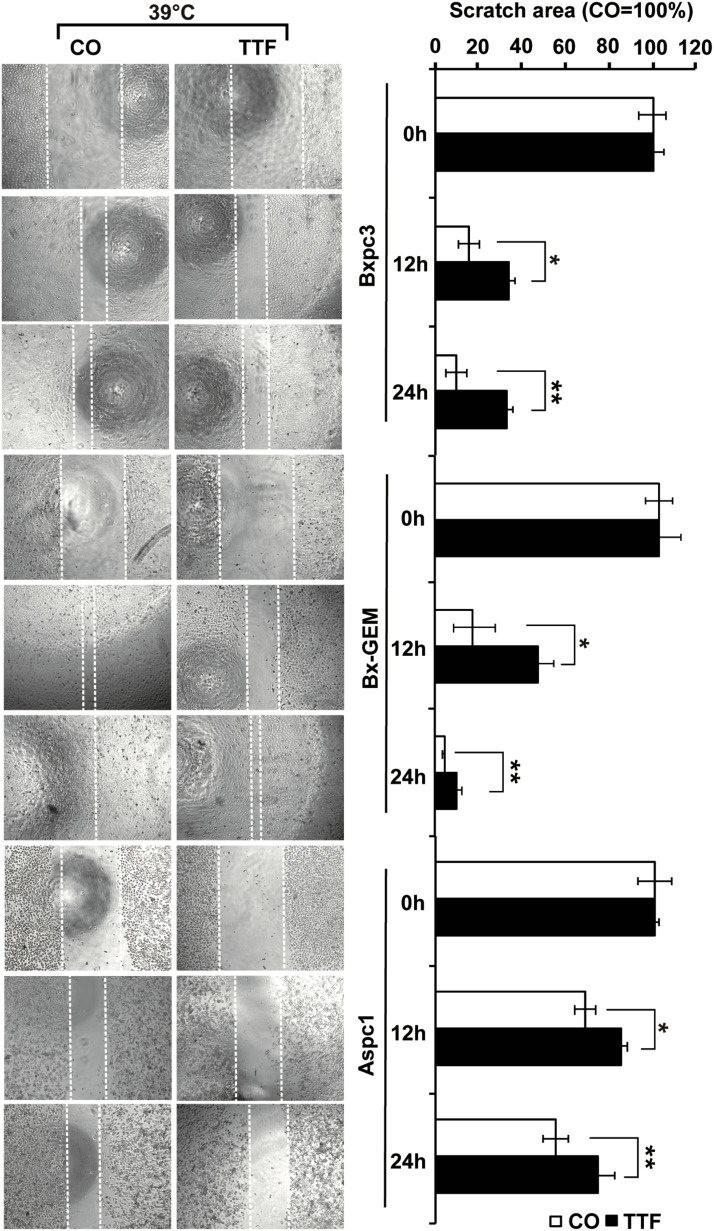
TTField-mediated inhibition of migration is stronger upon combination with hyperthermia. AsPC-1, BxPC-3 and Bx-GEM cells were seeded at a density of 4,000 cells/well in 96-well plates and grown to 90% confluency overnight. The cell layer was scratched with the tip of a 100-μL pipet, followed by treatment of the cells with TTFields at 38.5°C for 24 h. The controls (CO) were incubated at 38.5°C without exposure to TTFields. The closure of the wounded region was evaluated by microscopy at 0, 12 and 24 h after scratching. The gap width was measured using ImageJ. Representative images are shown on the left, and the means are shown in bar graphs on the right. The data are presented as the means ± SD. *P < 0.05, **P < 0.01.

The authors apologize for this error and state that this does not change the scientific conclusions of the article in any way.

