# Supplementary figures and images for: Establishment of Tumor Treating Fields Combined With Mild Hyperthermia as Novel Supporting Therapy for Pancreatic Cancer
Source: Front Oncol. 2021 Nov 3;11:738801. doi: 10.3389/fonc.2021.738801 (PMC8597267; doi:10.3389/fonc.2021.738801)

Crude Western blot films corresponding to Fig. 6C

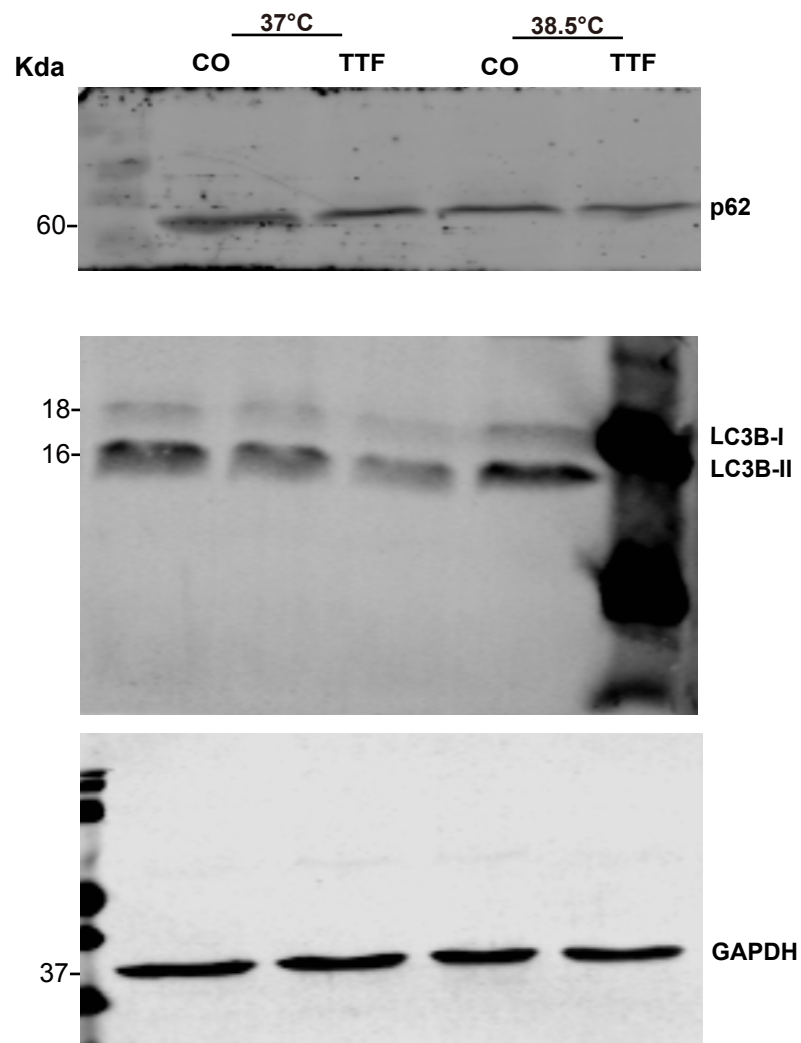

Supplement: Supplementary file 1 [file DataSheet_1.pdf]

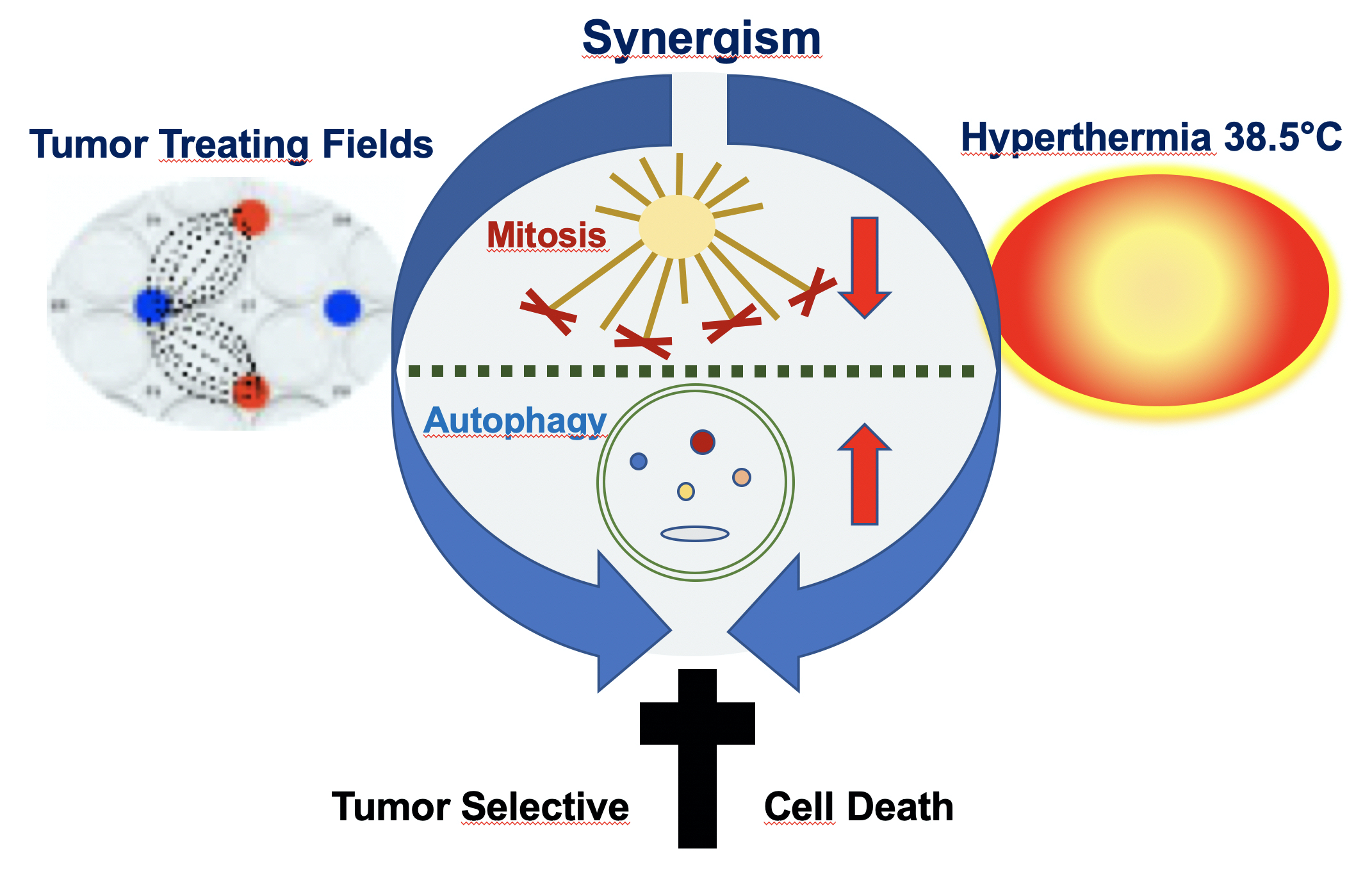

Supplement: Supplementary file 3 [file Image_1.jpeg]
